# Sequential Cranial and Overlooked Cervical Spine Injuries Due to Head Trauma: A Billiard-Like Mechanism

**DOI:** 10.7759/cureus.82292

**Published:** 2025-04-15

**Authors:** Hiroki Sato, Takeru Yokota, Kinshi Kato, Toshiki Sato, Yoshihiro Matsumoto

**Affiliations:** 1 Department of Orthopaedic Surgery, Minamisoma City General Hospital, Minamisōma, JPN; 2 Department of Orthopaedics, Fukushima Medical University School of Medicine, Fukushima, JPN; 3 Department of Orthopaedic Surgery, Fukushima Medical University School of Medicine, Fukushima, JPN

**Keywords:** alcohol use, cervical spine fracture, differential diagnosis, orthopedic opd cases, parathyroid hormone (pth)

## Abstract

A 55-year-old male construction worker presented with multiple fractures after falling downstairs and striking his forehead on a concrete wall, an incident preceded by consuming approximately 50 g of alcohol. Initial evaluation revealed a contaminated 7 cm forehead laceration and a depressed frontal bone fracture, with no neurological deficits or intracranial bleeding on computed tomography (CT), leading to discharge with follow-up instructions despite mild intoxication. The next day, neck pain prompted further imaging, uncovering a complex injury pattern: frontal bone fracture, occipital base fracture, C1 anterior arch fracture, and C4 vertebral body and spinous process fractures, with magnetic resonance imaging (MRI) excluding intervertebral disc injury. Conservative management with a Philadelphia collar for two months, followed by a soft collar for three months, and teriparatide to aid bone healing resulted in the patient returning to work symptom-free after six months. Follow-up imaging at one year (CT) showed no displacement and partial bone healing, while a three-year MRI confirmed no posttraumatic complications. This case illustrates a rare sequential fracture pattern from head trauma, resembling a billiard-like chain reaction where indirect axial force cascades through the frontal bone, occipital base, and cervical spine. Alcohol likely delayed pain recognition by elevating the pain threshold, highlighting the need for comprehensive cervical imaging in head trauma patients - especially those with intoxication or altered consciousness - to prevent missed diagnoses and ensure timely intervention.

## Introduction

Head trauma is a common clinical scenario that frequently results in associated cervical spine injuries (CSIs), yet these injuries are often underrecognized during initial evaluations due to their subtle presentation or the overshadowing focus on cranial injuries [1]. The cervical spine’s anatomical proximity to the skull and its biomechanical vulnerability make it susceptible to forces transmitted indirectly from head impacts, leading to a spectrum of injury patterns, ranging from ligamentous damage to complex fractures [2]. Among these, axial loading injuries - such as the well-documented Jefferson fracture of the atlas (C1) - are classically associated with head trauma, where force dissipates through the craniocervical junction [3,4]. However, less common sequential fracture patterns involving multiple cranial and cervical structures remain poorly understood and infrequently reported, often escaping detection unless comprehensive imaging is pursued [5]. These rare mechanisms can arise from a cascade of force transmission, where an initial impact triggers subsequent structural failures in adjacent or distant regions, mimicking dynamic physical phenomena such as a billiard-ball chain reaction [6]. Alcohol intoxication, a frequent cofactor in trauma cases, may further complicate early diagnosis by masking pain [7] or altering patient awareness [8], increasing the risk of missed injuries. We present a case of a 55-year-old male who sustained extensive fractures involving the frontal bone, occipital base, C1, and C4 vertebra following a fall downstairs that resulted in a direct forehead impact. This case exemplifies an unusual sequential fracture mechanism initiated by the primary cranial trauma, with axial forces radiating to the occipital bone and cervical spine in a stepwise fashion. By reporting this case, we aim to highlight the importance of thorough cervical assessment in head trauma patients and enhance the understanding of rare, force-transmitted fracture patterns, ultimately advocating for improved diagnostic vigilance to prevent delayed recognition and long-term complications.

## Case presentation

A 55-year-old male construction worker with no significant past medical history, including psychiatric disorders or prescription medications, presented to the emergency department of a rural Japanese hospital with no full-time orthopedic specialists following a traumatic fall. The patient maintained an independent lifestyle, with a documented history of intermittent alcohol use and no history of tobacco use. On the day of the injury (day X), he consumed approximately 50 g of alcohol-equivalent to 500 mL of beer and two shots of shochu diluted with water - before slipping on a staircase and striking his forehead against a concrete wall. He arrived at the emergency department with a 7 cm superficial laceration on his forehead, prompting immediate evaluation.

**Figure 1:**
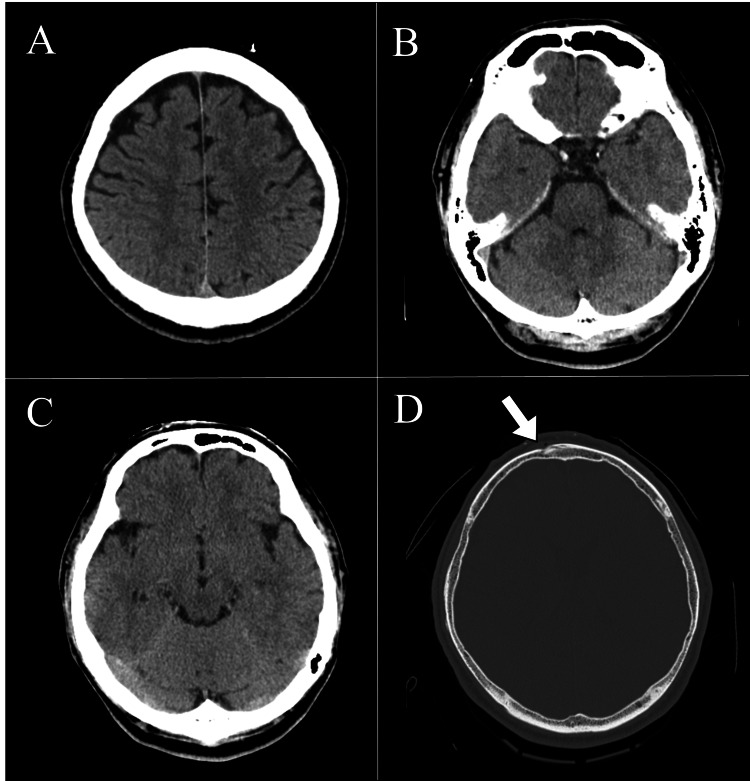
Head CT findings on the day of injury (day X). A–D represent axial images at different levels. No obvious hemorrhagic lesions were observed in the intracranial space on any of the sectional images. A: High parietal level. B: Pons level. C: Midbrain level. D: Fracture level, with the white arrow indicating the fracture site of the frontal bone.

Upon arrival on day X, the patient was assessed by a non-orthopedic physician. The patient’s vital signs were stable: body temperature was 35.9°C, systolic blood pressure was 137 mmHg, diastolic blood pressure was 69 mmHg, pulse rate was 89 bpm, oxygen saturation was 97%, and respiratory rate was 20 bpm. Despite mild intoxication, he remained fully alert with a Glasgow Coma Scale score of 15 (E4V5M6) and exhibited no neurological deficits or pain complaints. A computed tomography (CT) scan revealed a depressed fracture of the frontal bone but no evidence of intracranial bleeding (Figure 1).

**Figure 2:**
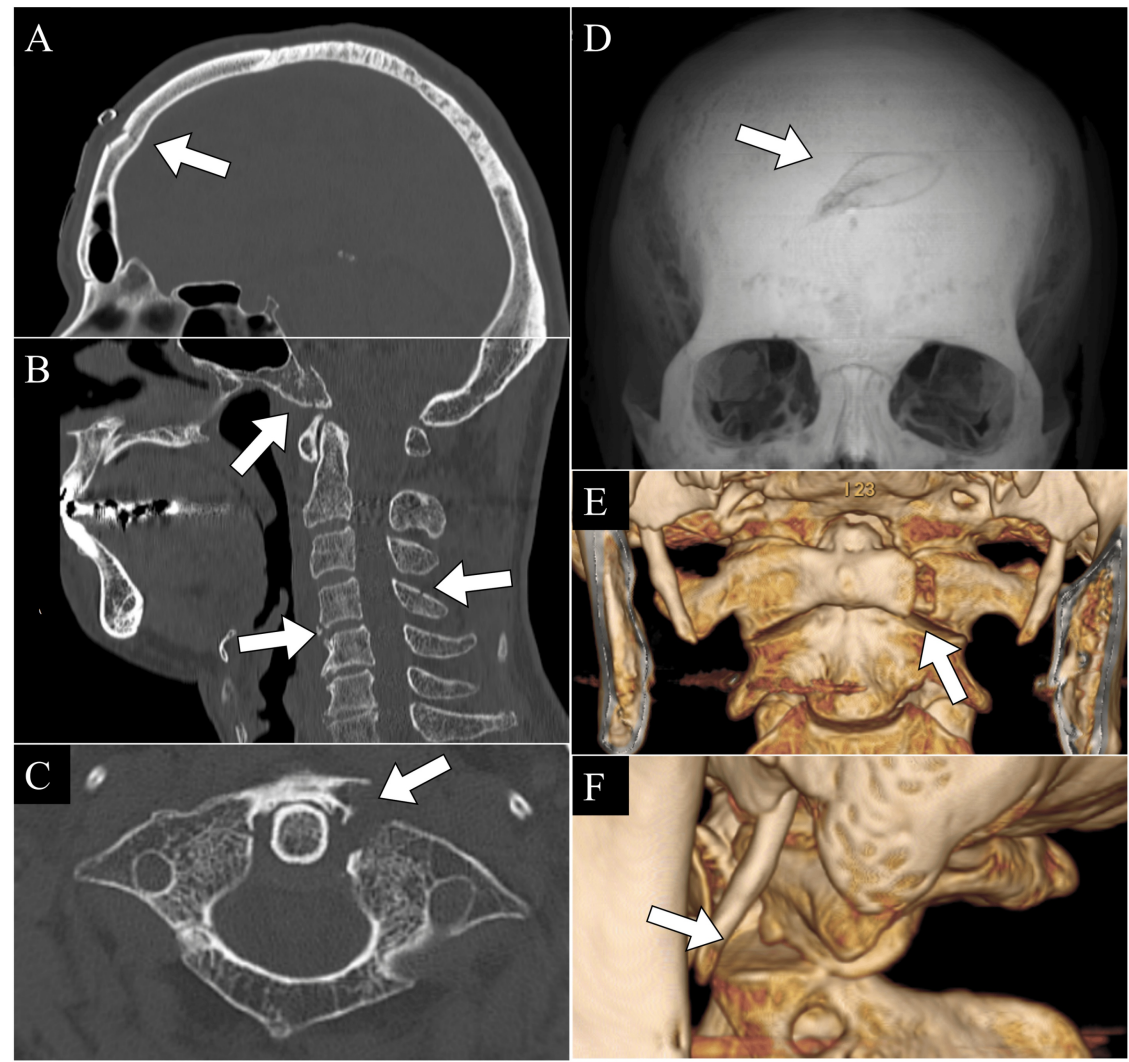
Head and cervical spine CT findings on the day following the injury (day X+1). A: Sagittal cut of the head CT, showing a fracture of the frontal bone. B: Sagittal cut of the cervical spine CT, revealing fractures of the occipital base, the vertebral body of C4, and the spinous process of C4. C: Axial cut of the cervical spine CT at the level of the atlas (C1), demonstrating a fracture of the anterior arch of the atlas with displacement. D: Three-dimensional reconstruction of the head CT, showing a fracture of the frontal bone. E: Three-dimensional reconstruction of the cervical spine CT, showing a fracture of the anterior arch of the atlas. F: Three-dimensional reconstruction of the cervical spine CT, revealing joint incongruity between the C1 and C2 vertebra. White arrows in A–F indicate the respective fracture sites.

**Figure 3:**
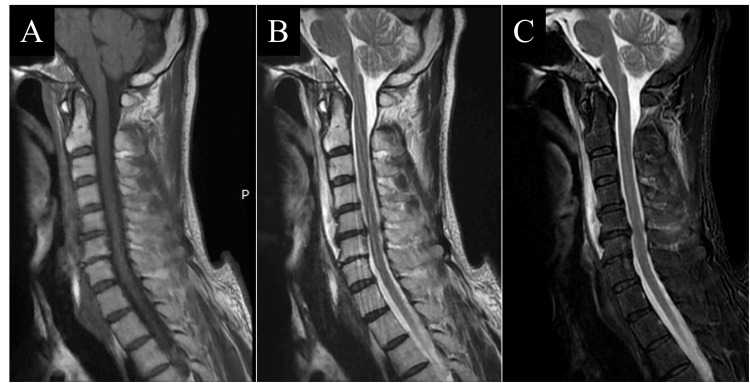
Cervical spine MRI on the day following the injury (day X+1). A: Sagittal T1-weighted image. B: Sagittal T2-weighted image. C: Sagittal short tau inversion recovery (STIR) image. In A–C, signal changes within the bone were observed at the occipital base, C4 vertebral body, and C4 spinous process. Similarly, hematoma formation was noted in the anterior longitudinal ligament in front of the cervical spine. However, no signal changes were observed within the intervertebral discs or the spinal cord.

After wound management and follow-up instructions for a neurology appointment, he was discharged. However, the following day (day X+1), the patient returned with new-onset neck pain, leading to a referral for orthopedic evaluation. Additional imaging uncovered a complex injury pattern: fractures of the occipital base, the anterior arch of C1, the C4 vertebral body, and spinous process, in addition to the previously identified frontal bone fracture (Figure 2). Magnetic resonance imaging (MRI) confirmed the absence of intervertebral disc injury (Figure 3). As there were no subsequent falls after day X, these injuries were attributed to the initial trauma, yielding final diagnoses of (1) frontal bone fracture, (2) occipital base fracture, (3) C1 anterior arch fracture, and (4) C4 vertebral body and spinous process fractures.

**Figure 4:**
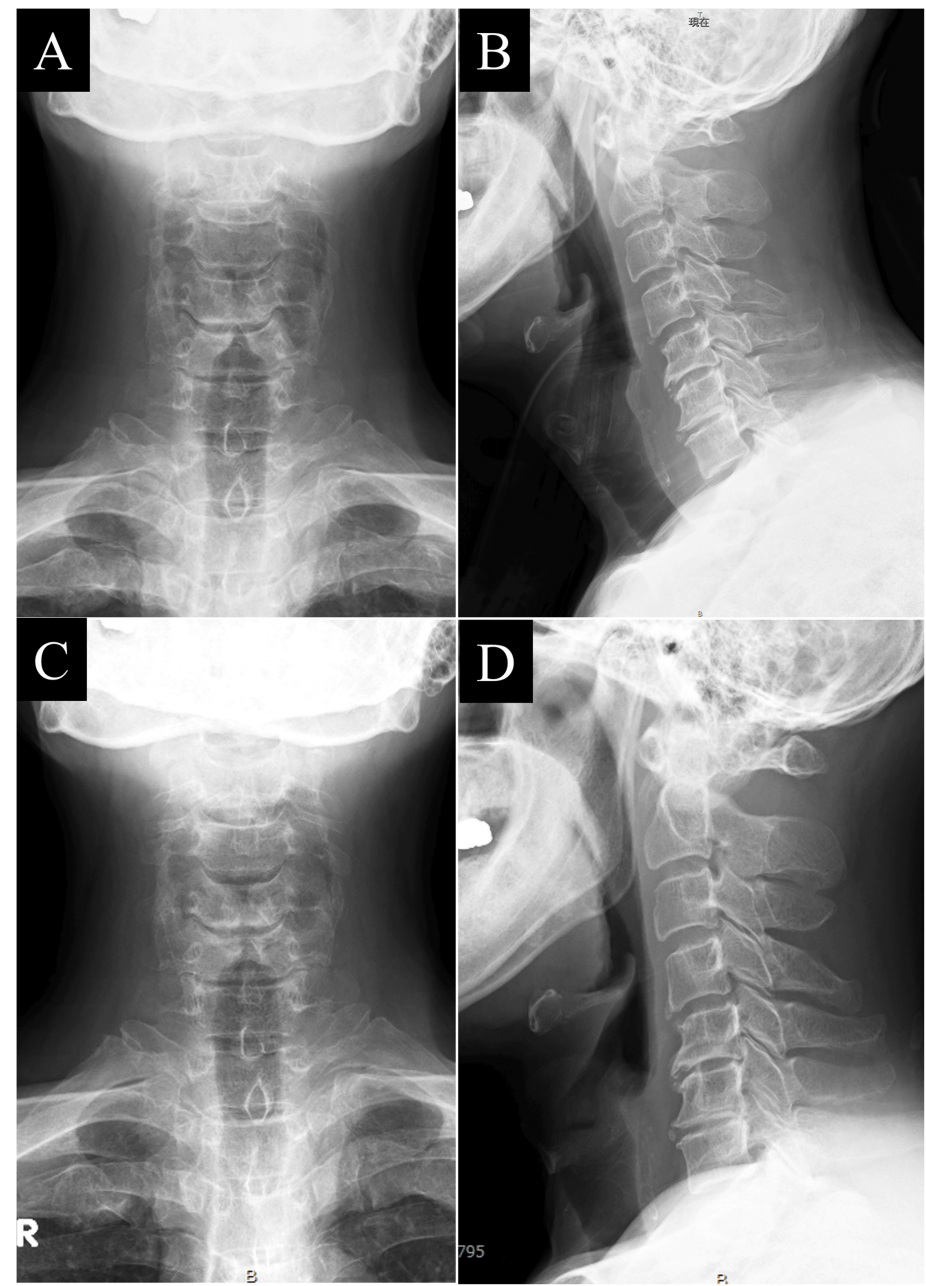
Plain radiographs of the cervical spine at the time of injury and six months post-injury. A: Plain radiograph at the time of injury. B: Plain radiograph at six months post-injury. No displacement of the fracture sites was observed from A to B.

**Figure 5:**
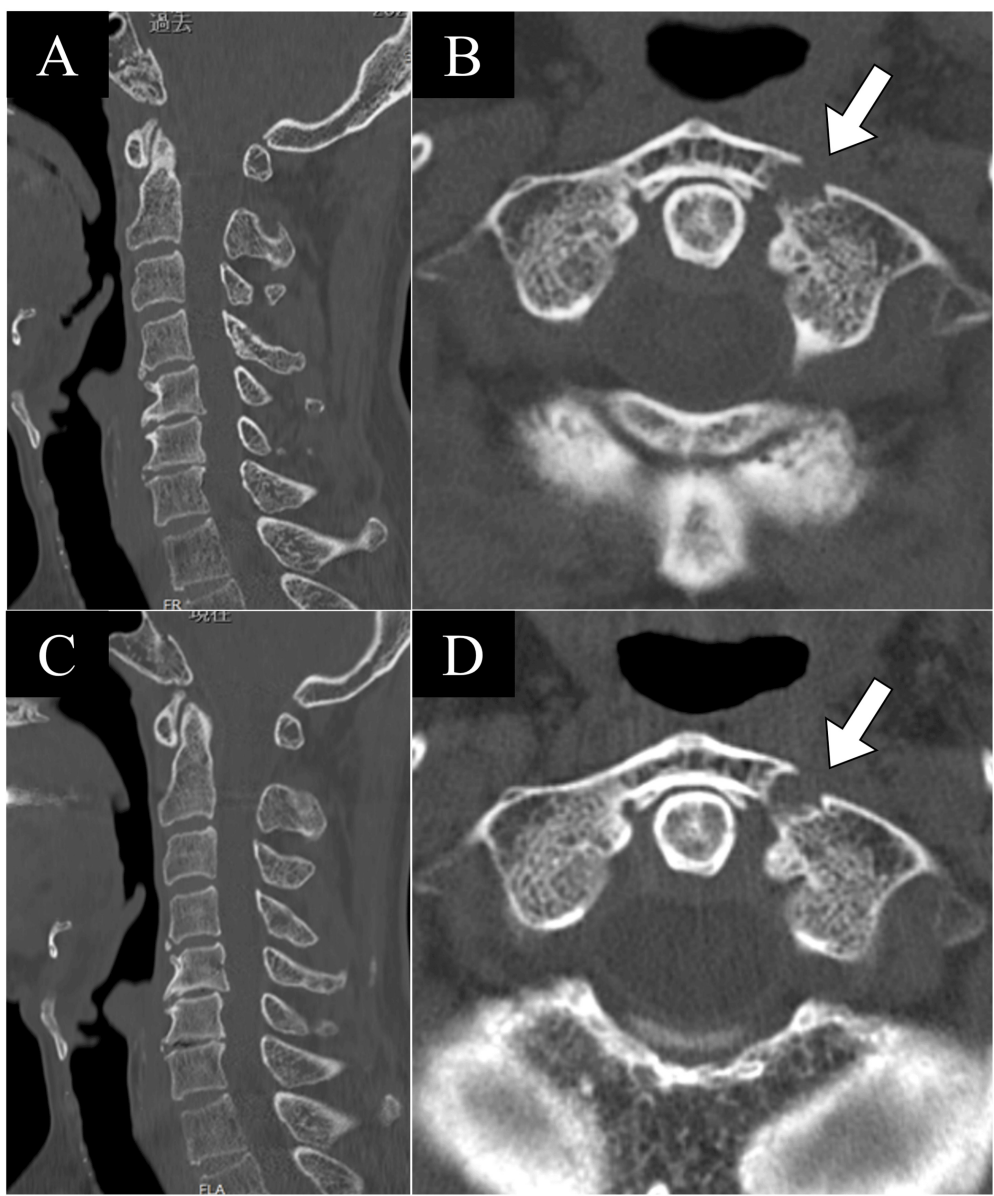
Cervical spine CT at four months and one year post-injury. A, B: Cervical spine CT at four months post-injury. A: No displacement was observed in the occipital bone or C4 vertebra. B: Callus formation was confirmed at the fracture site of the atlas (C1). C, D: Cervical spine CT at a year post-injury. C: No displacement of the C4 vertebra was observed, and bone healing was achieved in the occipital bone. D: Bony bridging was confirmed at the fracture site of the atlas (C1), indicating partial bone healing.

**Figure 6:**
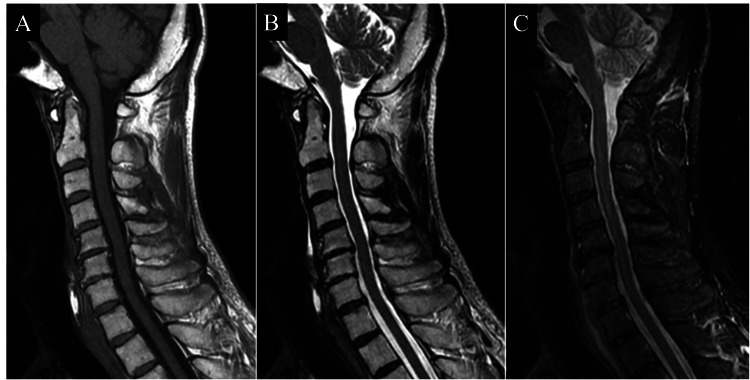
Cervical spine MRI at three years post-injury. A: Sagittal T1-weighted image. B: Sagittal T2-weighted image. C: Sagittal short tau inversion recovery (STIR) image. In A–C, the signal changes associated with traumatic changes at the time of injury observed have resolved.

Conservative management was selected for all fractures. The frontal bone fracture was rigorously monitored for potential neurological complications, while the cervical spine injuries were stabilized with a Philadelphia collar for two months, followed by a soft collar for an additional three months. To enhance bone healing, daily subcutaneous teriparatide (Forteo, Eli-Lilly, Indianapolis, IN) was administered [9]. At three months post-injury, imaging showed no fracture displacement (Figure 4A), and collar therapy was completed. By four months, follow-up CT confirmed stable occipital bone and C4 fractures with no displacement, alongside callus formation at the C1 fracture site (Figures 5A, 5B), prompting discontinuation of teriparatide. At six months, the patient reported no neck pain, and radiography revealed no fracture displacement (Figure 4B), allowing him to resume work. One year post-injury, he remained free of neck pain or neurological symptoms; CT demonstrated consolidation of the occipital bone and no displacement of the C4 fractures, with bony bridging indicating partial healing at C1 (Figures 5C, 5D). A three-year follow-up MRI revealed no signs of nonunion, degenerative changes, or alignment issues (Figure 6), concluding the patient’s care with a full recovery.

## Discussion

Herein, we present a rare case of sequential cranial and cervical fractures resulting from head trauma. Multiple CSIs can occur in patients with head trauma via a billiard-like mechanism [6]. In this particular case, despite the patient consuming alcohol, there was a full Glasgow Coma Scale (GCS) score and an absence of immediate symptoms. The diagnosis of cervical spine fractures was initially delayed, potentially leading to neurological symptoms or long-lasting sequelae. This underscores the critical importance of thorough CSI evaluation during the initial management of patients with head trauma. This case serves as a stark reminder of the complexities in head trauma assessment, particularly when confounding factors such as alcohol are involved.

In this case, in addition to cranial fractures, multiple fractures were observed, including those of the frontal bone, occipital skull base, atlas, and lower cervical vertebrae. Previous studies have reported that multiple cervical fractures occur in 33-36.1% of cervical spine fracture patients with head trauma [1]. Furthermore, fractures of the posterior central skull base were observed in this case, while fractures of the middle central skull base were reported to occur in 20% of cases involving skull base fractures [10]. The coexistence of multiple craniomaxillofacial fractures with both CSIs and head injuries is rare [11]. In this case, the frontal bone fracture was likely caused by direct trauma to the head, while subsequent indirect forces may have led to fractures of the occipital skull base, atlas, and cervical vertebra 4 in a sequential manner. The imaging findings provided in this case report demonstrate that these fractures occurred as a series of injuries caused by a single traumatic event.

Special attention must be paid to CSIs in patients with head trauma, as such injuries can result in new neurological symptoms and severe sequelae. Furthermore, head trauma necessitates careful evaluation for CSIs due to their potential severity [12]. Previous studies have reported that 4.76% of patients with head trauma have CSIs, and in cases involving head trauma with impaired consciousness, the incidence of CSIs is relatively high at 6.7% [13]. Conversely, 35% of patients with CSIs or spinal cord injuries have been reported to experience moderate to severe head trauma, indicating a strong association between head trauma and CSIs [14].

Head trauma is a common occurrence, with an estimated 300,000 cases occurring annually in Japan. In the United States, traumatic brain injury occurs in 1.5 million cases annually [15]. Physicians managing patients with head trauma in emergency departments must recognize the potential for CSIs associated with head trauma. In this case, although the patient presented with a frontal bone fracture due to head trauma, the CSIs should have been assessed during the initial evaluation, regardless of the presence or absence of symptoms.

Several criteria exist for evaluating CSI risk. Prominent examples include the National Emergency X-Radiography Utilization Study (NEXUS) Low-Risk Criteria [16] and the Canadian C-Spine Rule (CCR) [17]. The NEXUS criteria indicate that patients who meet all five conditions - absence of posterior cervical tenderness, no focal neurological deficits, normal level of consciousness, no evidence of intoxication, and no painful distracting injuries - are at a low risk of CSI. The CCR states that high-risk patients, as identified by factors such as age, mechanism of trauma, or sensory abnormalities, should undergo radiographic evaluation, whereas patients with five low-risk factors can be assessed for cervical spine range of motion. Patients able to rotate their necks by 45° to either side do not require radiographic imaging, regardless of pain. In addition, Inagaki et al. proposed new rules for evaluating CSIs using CT. They recommend CT for patients with a GCS < 14, cervical tenderness, neurological deficits, or specific mechanisms of trauma, such as falls from stairs, motorcycle accidents, or falls from heights [18].

If these criteria were applied in this case, it is probable that CSIs might have been detected during the initial evaluation. Despite alcohol consumption, he exhibited a full GCS score and showed no immediate symptoms. However, the delayed diagnosis of CSIs in this case was likely influenced by alcohol consumption, which may have contributed to altered consciousness and modified pain thresholds. The frequency and amount of alcohol use can affect pain perception [7], and alcohol has been reported to increase pain thresholds owing to its general pharmacological effects [19]. Furthermore, epidemiological studies have indicated that alcohol use is associated with delayed diagnosis of CSIs [20]. In cases where alcohol use may obscure pain symptoms, a thorough evaluation of trauma is required with greater attention than usual.

## Conclusions

This case report details a 55-year-old man with multiple fractures following a head injury, undetected initially owing to delayed neck pain and alcohol intoxication. It emphasizes the imperative for systematic cervical spine evaluation in head trauma, given that routine cranial imaging may fail to identify such injuries, particularly in patients with risk factors such as intoxication. Comprehensive CT or MRI assessment is vital to avert complications. This case supports the adoption of heightened clinical vigilance and standardized imaging protocols to improve diagnostic precision and patient outcomes.
